# Zeolite materials with Ni and Co: synthesis and catalytic potential in the selective hydrogenation of citral

**DOI:** 10.3762/bjnano.16.40

**Published:** 2025-04-14

**Authors:** Inocente Rodríguez-Iznaga, Yailen Costa Marrero, Tania Farias Piñeira, Céline Fontaine, Lexane Paget, Beatriz Concepción Rosabal, Arbelio Penton Madrigal, Vitalii Petranovskii, Gwendoline Lafaye

**Affiliations:** 1 Instituto de Ciencia y Tecnología de Materiales (IMRE), Universidad de La Habana, Zapata y G, 10400, La Habana, Cuba; 2 Universidad de las Ciencias Informáticas, Carretera a San Antonio km 21/2, 19370, La Habana, Cuba; 3 Université de Poitiers, CNRS, Institut de Chimie des Milieux et Matériaux de Poitiers (IC2MP), 4 rue Michel Brunet, Poitiers, France; 4 Facultad de Física, Universidad de La Habana, San Lázaro y L, 10400, La Habana, Cuba; 5 Centro de Nanociencia y Nanotecnología (CNyN), Universidad Nacional Autónoma de México (UNAM), 22860, Ensenada, B.C., México

**Keywords:** citral hydrogenation, cobalt–nickel mixture, impregnation, ion exchange, natural zeolite

## Abstract

Zeolitic materials incorporating mono- and bimetallic systems of nickel and cobalt were obtained from natural zeolite modified with Ni^2+^ and Co^2+^ chloride solutions through traditional ion exchange (IE) and impregnation (Imp) processes. Special attention was given to analyzing the cationic and anionic composition of the resulting materials. The catalytic potential was evaluated in the selective hydrogenation of citral, focused on the formation of unsaturated alcohols. The IE process replaced mainly Ca^2+^ and Na^+^ with Ni^2+^ and Co^2+^ cations in the zeolite phases (clinoptilolite and mordenite mix), while Imp resulted in higher metal content (2.0–2.7%) but retained significant amounts of chloride (1.9–3.8%), as confirmed by XRD and temperature-programmed reduction. The materials prepared by IE had negligible chloride content (0.02–0.07%), and their specific surface areas (138–146 m^2^/g) were greater than those of the materials obtained by Imp (54–67 m^2^/g). The bimetallic systems exhibited enhanced reducibility of the Co^2+^ and Ni^2+^ isolated cations, attributed to synergistic interactions that weakened the cation–framework binding. Catalytic activity tests showed that nickel species were primarily responsible for citronellal formation. Among all materials, the bimetallic CoNi_IE_ catalyst, prepared by IE, was the only one to produce unsaturated alcohols, suggesting that synergistic Ni–Co interactions played a role in their formation.

## Introduction

Numerous publications in the literature highlight zeolites modified with metallic species for various applications, leading to the invention of new functional materials for sustainable development, such as catalysts [1–3]. Among the various methods used to modify zeolites, ion exchange is the most widely employed. Different ion exchange methods are known, such as ion exchange in conventional solutions, in the solid phase with molten salts, and with gaseous phases. Depending on the chosen modification method, particularities regarding the elemental composition and application of the resulting materials occur [4–6]. Cu–Y zeolites were obtained by contacting Na–Y zeolite and Cu(II) nitrate solution, using two different methods, namely, conventional solution ion exchange and incipient wetness impregnation, followed by calcinations at 600 °C in air [5]. The authors reported that, among both zeolites, the Cu–Y material obtained by impregnation followed by calcination exhibited a higher surface area and pore volume, which can positively influence its potential application as a material to reduce greenhouse gas emissions.

While most studies focus on monocationic exchange, multicationic exchange has raised significant interest. The synergy of properties in multicationic systems enables the creation of materials with enhanced properties compared to monocationic zeolites, which is crucial for the development of advanced catalysts and other materials [7–10]. Natural zeolites attract significant attention because of their abundance, low cost, non-toxic nature, and other valuable physical and chemical properties.

Modifying these materials with inexpensive metals leads to low-cost catalysts for various different processes. The selective hydrogenation of α,β-unsaturated aldehydes, such as citral, to unsaturated alcohols is a crucial reaction for producing fine chemicals, fragrances, and other high-value products [11]. The catalysts preferred for this reaction are currently based on noble metals because of their excellent performance [12–13]. However, their high cost and scarcity requires strategies to reduce their use or replace them with non-noble alternatives offering comparable catalytic properties.

Despite extensive research on zeolite-supported transition metal catalysts, their application to citral hydrogenation remains very little explored [11]. Most recent studies focus on the selective hydrogenation of related biomass-derived compounds, such as furfural and cinnamaldehyde [3,14–15]. Zeolites modified with nickel and cobalt have shown promising results in selective hydrogenation reactions, owing to their high dispersion of active sites and tunable acidity. For instance, a zeolite-supported Ni catalyst has demonstrated selectivity in furfural hydrogenation by leveraging controlled acidity to prevent overhydrogenation and optimize product yields [15]. Similarly, Co-modified zeolites have been effectively used in cinnamaldehyde hydrogenation, where the balance between acidity and metal dispersion facilitates the selective reduction of the carbonyl group while preserving other reactive sites [3].

Building on these insights, this work presents research on the selective hydrogenation of citral using both monometallic and bimetallic nickel and cobalt catalysts supported on natural zeolite, which was modified with Ni^2+^ and Co^2+^ chloride solutions through traditional ion exchange (IE) and impregnation (Imp) processes. Emphasis is put on analyzing the cationic and anionic composition of the materials resulting from both methods and the catalytic performance in citral hydrogenation with a focus on enhancing the formation of unsaturated alcohols.

## Results and Discussion

### Composition and characterization of the materials

**Figure 1:**
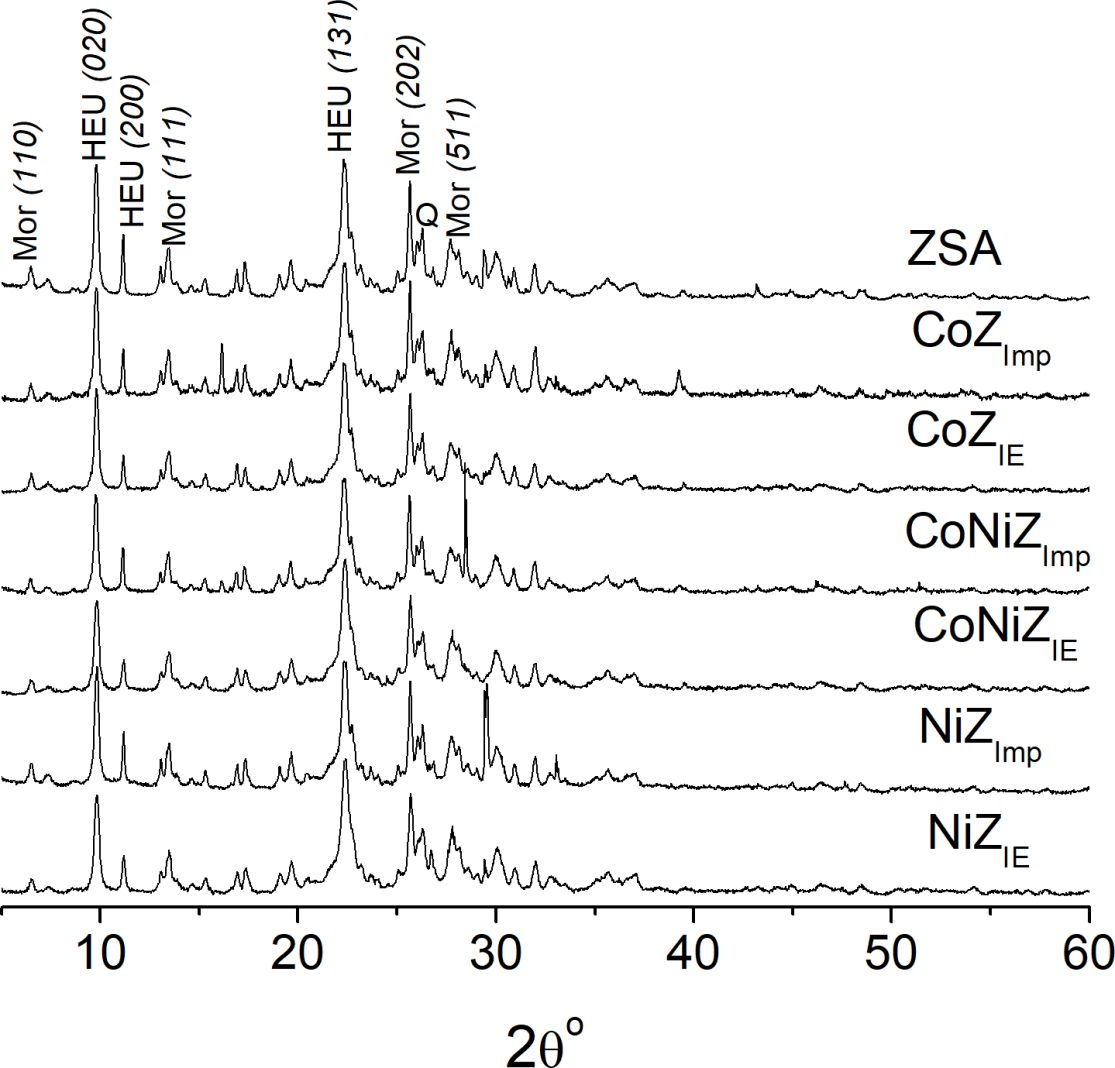
XRD pattern of the starting zeolite mineral (ZSA) and the materials obtained through traditional ion exchange (IE) and impregnation (Imp) methods. The Miller indexes (*hkl*) corresponding to the zeolite phases are shown in the pattern of the ZSA. Mor, HEU, and Q are associated to the phases of mordenite, clinoptilolite–heulandite, and quartz, respectively.

**Figure 2:**
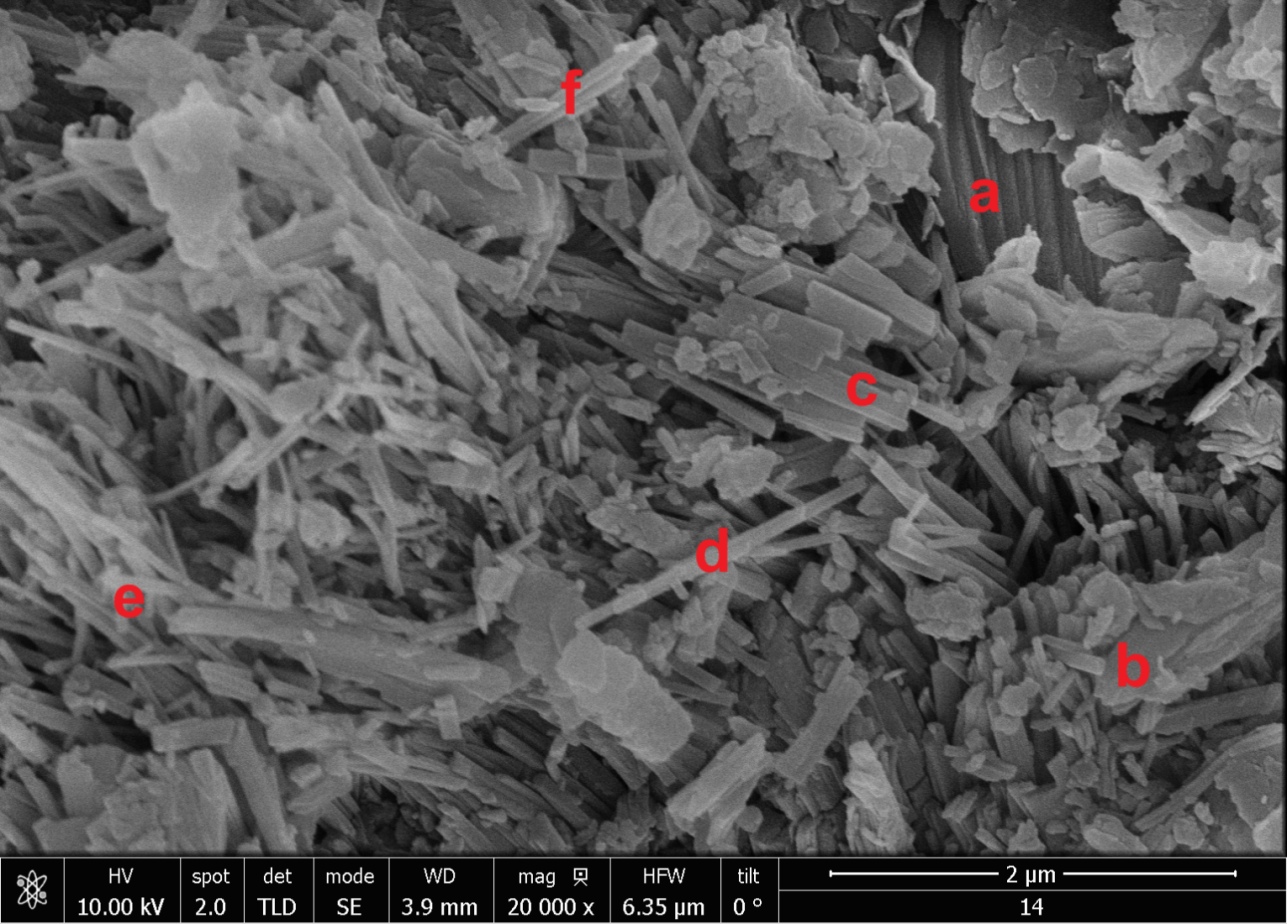
SEM micrograph of ZSA from the San Andrés deposit. Some HEU crystals are indicated by a, b, and c, where a and b exhibit tabular characteristics while c has slat morphology. Mordenite crystals, marked by d, e, and f, show acicular to fibrous shapes.

XRD patterns and a SEM micrograph of the starting zeolite mineral (ZSA) are shown in Figure 1 and Figure 2, respectively. The diffraction patterns are normalized and evidence the presence of mordenite and clinoptilolite–heulandite-type zeolites through their main diffraction peaks indicated on the ZSA graph. Other minor phases such as quartz are also present.

The SEM image shows a variety of crisscross crystals, which have the morphology expected for the zeolite types evidenced by XRD [16–17]. Very elongated crystals with acicular to fibrous characteristics, associated with mordenite, can be observed. Additionally, clinoptilolite–heulandite crystals with slats and tabular morphology are present. The amounts of clinoptilolite–heulandite and mordenite crystals displayed in the SEM image correspond to the intensity order of these zeolites shown in the XRD pattern (Figure 1). This indicates that zeolitic mineral from the San Andrés deposit is mainly composed of a mordenite and clinoptilolite–heulandite mixture. Furthermore, the SEM micrographs reveal free spaces between crystallites, contributing to the material’s porosity and mesoporosity.

The XRD patterns of the materials obtained using both Imp and IE showed that the framework of both zeolites remains largely unchanged after the applied treatments. However, the materials obtained by Imp exhibit new peaks, such as those observed at 16.1° and 39.3° in CoZ_Imp_, at 29.5° in NiZ_Imp_, and at 28.5° in CoNiZ_Imp_. These peaks are attributed to impurities, such as mixed metal chloride salts, deposited on the zeolite surface. Specifically, the peaks at 16.1° and 39.3° correspond to both cobalt(II) chloride (CoCl_2_·2H_2_O, card 96-900-9874) and cobalt(II) hydroxychloride (Co_2_Cl(OH)_3_, card 96-231-0849). The diffraction peak at 29.5° is associated with NiCl_2_ (card 96-900-9133), but it could also be assigned to FeCl_2_ (lawrencite, card 96-901-9129) or CaCl_2_·2H_2_O (sinjarite, card 96-100-1836). The peak at 28.5° can be related to FeCl_2_·2H_2_O (card 96-231-0808), NaCl (halite, card 96-900-0630), or CaCl_2_O_4_ (calcium hypochlorite, card 96-220-7380). The low nickel and cobalt contents in the CoNiZ_Imp_ material (Table 1) may limit the detectability of diffraction peaks associated to cobalt and nickel chloride salts on the zeolite support.

**Table 1:** Cobalt and nickel contents and Si/Al ratio values for the materials obtained through IE and Imp.

Sample	Co, wt %	Ni, wt %	Si/Al

CoZ_IE_	1.54 ± 0.08	—	5.47
NiZ_IE_	—	1.29 ± 0.1	5.20
CoNiZ_IE_	0.97 ± 0.07	1.00 ± 0.08	5.47
CoZ_Imp_	2.56 ± 0.20	—	5.35
NiZ_Imp_	—	2.71 ± 0.21	5.22
CoNiZ_Imp_	1.16 ± 0.08	0.87 ± 0.06	5.17
ZSA	—	—	5.20

**Figure 3:**
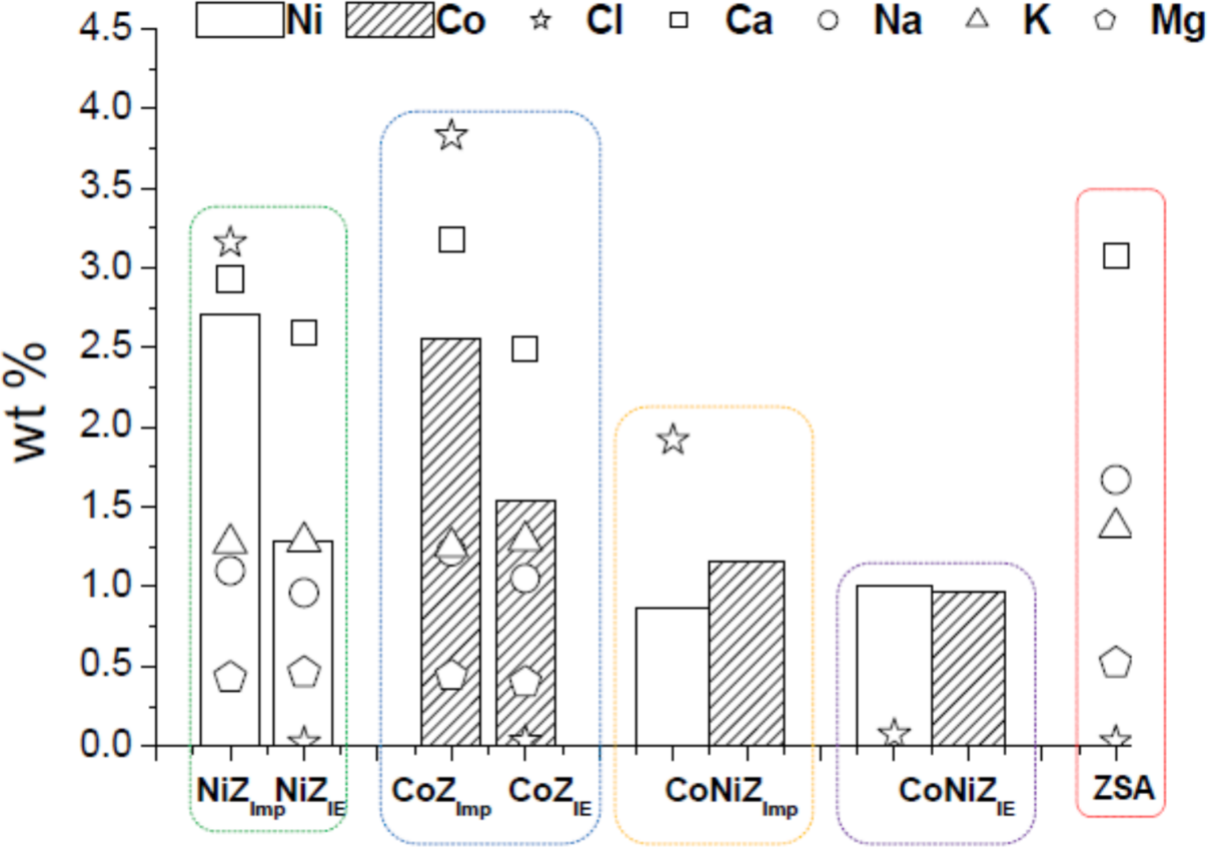
Elemental composition determined by X-ray fluorescence of the materials obtained by IE and Imp.

The contents of cobalt and nickel and other elements, as well as the Si/Al ratios for the obtained materials are shown in Table 1 and/or Figure 3. The Si/Al ratio shows no significant change (Table 1), indicating a high degree of stability for the clinoptilolite–heulandite and mordenite phases after the applied hydrothermal treatments. Additionally, it can be observed that the Ni and Co concentrations are generally higher in materials obtained through Imp compared to those produced via IE. However, it is important to note that materials obtained by Imp tend to have high chloride contents, whereas those produced by IE exhibit negligible chloride levels (Figure 3). The chloride originates from the NiCl_2_ and CoCl_2_ solutions used in the treatments, which remain as residues on the materials. The residual solution may be retained through surface adsorption within the porosity and mesoporosity formed between the zeolite crystallites, as shown in the SEM image (Figure 2). To remove excess solution, water washes are typically applied. The materials obtained via IE underwent extensive washing with distilled water, while those obtained via Imp were only lightly washed, leading to the observed differences in chloride content. According to this, there are color differences between the obtained materials and the starting zeolitic mineral (see Figure S1 of Supporting Information File 1), which are stronger in the materials obtained by Imp due to their higher content of residual salts.

Figure 3 also presents the Na, K, Ca, and Mg contents for the monometallic materials obtained from both treatments. Despite the previously mentioned observations, both types of materials (from IE and Imp) exhibit a reduction in Na, K, Ca, and Mg levels compared to ZSA, with this decrease being more pronounced in the materials obtained via IE. This suggests that in both treatments an ion exchange process occurred between the Ni^2+^ and Co^2+^ cations from solutions and the Ca^2+^, Na^+^, K^+^, and Mg^2+^ cations from the zeolite phases (mordenite and clinoptilolite-heulandite) contented within ZSA, as represented by Equation 1:


[1] $${\text{A}}_{2}^{n+}-{\text{Z}}_{\left(\text{s}\right)}+n{\text{M}}_{\left(\text{aq}\right)}^{2+}={\text{M}}_{n}^{2+}-{\text{Z}}_{\left(\text{s}\right)}+2{\text{A}}_{\left(\text{aq}\right)}^{n+},$$

where Z_(s)_ is the solid zeolite phase, and A*^n^*^+^ denotes the Ca^2+^, Na^+^, K^+^, and Mg^2+^ cations within the zeolite. M^2+^_(aq)_ represents the Ni^2+^ and Co^2+^ cations from the solution.

Table 2 presents the quantities of Co^2+^, Ni^2+^, and Cl^−^ in equivalent moles per 100 g of each material, as well as the equivalent mole ratio of Cl^−^ anions relative to Co^2+^, Ni^2+^, and the Co^2+^–Ni^2+^ mixture. These values were determined from the elemental contents reported in Table 1 and Figure 3. It is evident that the equivalent mole ratios are different from unity, which suggests that these ions are not totally neutralizing their charges (i.e., 2 equivalents of Cl^−^ per 1 equivalent of metal cation (see M^2+^ in Equation 1)). Moreover, in most cases they are less than unity, which indicates that a portion of the Co^2+^ and Ni^2+^ cations have not neutralized their positive charges with Cl^−^ anions. Instead, these cations likely neutralize their charge with negative charges from the zeolite framework, suggesting they occupy extra-framework cationic positions as compensation cations. This observation aligns with the earlier discussion that an ion exchange process occurred between these metal cations and the cations from the zeolite phases (as represented in Equation 1). For samples obtained by impregnation, the equivalent mole ratios are much higher mainly because of the large amount of chloride remaining on the surface. However, this does not mean that there are no Ni^2+^ and Co^2+^ cations occupying exchange positions. Note that chloride can also form salts with iron, sodium, and calcium cations leaving the zeolite, as proven by XRD.

**Table 2:** Equivalent mole values of Co^2+^, Ni^2+^, and Cl^−^ per 100 g of material (equiv/100 g) and the equivalent mole ratio (Cl^−^/M^2+^) of Cl^−^ anions relative to metal cations (Co^2+^, Ni^2+^, and Co^2+^–Ni^2+^ mixture) of materials obtained by IE and Imp.

Sample	Co^2+^, equiv/100 g	Ni^2+^, equiv/100 g	Cl^−^, equiv/100 g	Cl^−^/M^2+^

CoZ_IE_	0.052 ± 0.003	—	8.60 ± 0.47 × 10^−4^	0.0165
NiZ_IE_	—	0.0438 ± 0.0033	5.69 ± 0.34 × 10^−4^	0.0130
CoNiZ_IE_	0.032 ± 0.0023	0.034 ± 0.0027	1.90 ± 0.13 × 10^−4^	0.0287
CoZ_Imp_	0.086 ± 0.0067	—	0.1080 ± 0.0097	1.2558
NiZ_Imp_	—	0.092 ± 0.0071	0.0891 ± 0.0071	0.9684
CoNiZ_Imp_	0.039 ± 0.0026	0.029 ± 0.002	0.0541 ± 0.0037	0.7955

In line with expectations for a natural zeolite, the studied materials exhibited hybrid type-I/IV adsorption isotherms (see Figure S2 in Supporting Information File 1), indicating the presence of both microporous and mesoporous structures. The isotherms showed a hysteresis loop, which is associated with capillary condensation within the materials’ pores and is expected to be more pronounced in HEU than in Mor. This can be attributed to the smaller channel diameter of HEU (maximum opening of 0.31 × 0.75 nm^2^) compared to Mor (maximum opening of 0.70 × 0.65 nm^2^). Additionally, the kinetic diameter of a nitrogen molecule (0.36 nm) is closer to the HEU channel diameter, further influencing the observed behavior.

Table 3 displays the specific surface area (SSA) and microporous volume (*V*_micro_) values determined for the obtained materials. It is evident that all materials acquired through Imp have lower SSA and *V*_micro_ values compared to those obtained via IE. This reduction is attributed to the presence of more adsorbed salts (chlorides) on the materials’ surface, which is in agreement with the XRD results.

**Table 3:** Specific surface area (SSA) and microporous volume (*V*_micro_) of the ZSA and the materials obtained using IE and Imp.

Sample	ZSA	CoZ_IE_	CoZ_Imp_	NiZ_IE_	NiZ_Imp_	CoNiZ_IE_	CoNiZ_Imp_

SSA (m^2^/g)	134	138	67	146	54	142	64
*V*_micro_ (cm^3^/g)	0.0316	0.0326	0.0015	0.0385	0.0067	0.0439	0.0139

Conversely, the SSA and *V*_micro_ values for materials obtained by IE are higher than those of ZSA. This increase is consistent with expectations [17–18], as the ion exchange and water washing treatments effectively clean the materials’ surface, thereby enhancing the available surface area and porosity (microporosity) for adsorption.

**Figure 4:**
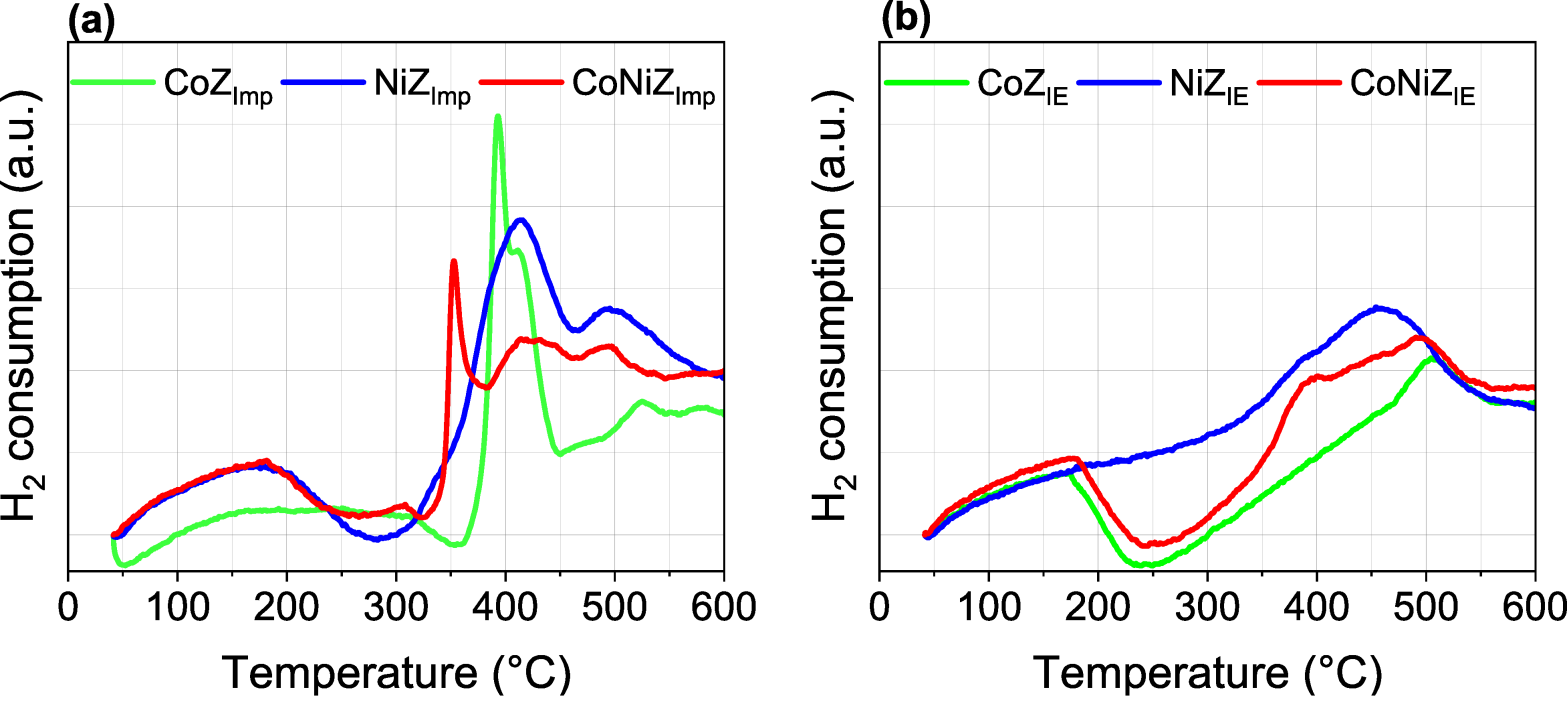
TPR profiles for the mono- and bimetallic materials obtained by (a) Imp and (b) IE.

The temperature-programmed reduction (TPR) profiles of the materials obtained by both methods are shown in Figure 4. These profiles vary significantly depending on the used modification method. It has been reported that the cations supported on zeolites can be thermally reduced by hydrogen, which is influenced by the nature of co-cations present [19–22]. The TPR profiles display hydrogen consumption peaks at different temperatures, corresponding to the reduction of the nickel and cobalt cations arranged on the zeolite support. The TPR profiles of materials prepared via Imp show a greater number of hydrogen consumption peaks compared to those obtained by IE. Two peaks appeared centered at 170 °C and 500 °C for CoZ_IE_, while CoZ_Imp_ exhibits hydrogen consumption in the 150–350 °C range and three additional peaks centered at 390 (intense), 410, and 515 °C. Similarly, NiZ_IE_ exhibits two peaks at 380 (low) and 450 °C, whereas NiZ_Imp_ displays three peaks at 150, 410, and 500 °C. Regarding the bimetallic systems, CoNiZ_IE_ shows three peaks at 170, 380, and 500 °C, while CoNiZ_Imp_ shows five peaks at 170, 310, 350 (intense), 420, and 500 °C. In line with the elemental composition (Figure 3, Table 1 and Table 2), the textural parameters (Table 2) and previous discussions, hydrogen consumptions peaks can be attributed to the reduction of cobalt and nickel cations present as both isolated cations (compensation cations in extra-framework ionic positions) and chloride salts adsorbed on the zeolitic support. This distinction explains the differences between the TPR profiles. Thus, materials obtained by IE exhibit peaks associated only with isolated cations, while materials obtained by Imp show peaks linked to both isolated cations and chlorides.

Careful analysis of the profiles suggests that isolated Co^2+^ cations in extra-framework ionic positions undergo two hydrogen consumption events for their complete reduction, that is, one at 170 °C and another one in the 500–515 °C region. Similarly, isolated Ni^2+^ cations in these positions experience two reduction events, namely, one at 380 °C and another one in the 450–500 °C region. This behavior is consistent across both modification methods and is observed in both monometallic and bimetallic systems. Beside this, the reduction of isolated cations is thermally favored in the bimetallic systems. Note that the intensity of the 380 °C peak, associated with isolated Ni^2+^ cation reduction, increases in bimetallic CoNiZ_IE_ compared to monometallic NiZ_IE_. Furthermore, the reduction of isolated Co^2+^ cations that takes place at 510 °C in the monometallic CoZ_IE_ is shifted to a lower temperature (500 °C) in the bimetallic CoNiZ_IE_. This suggests a mutual synergistic influence between Ni^2+^ and Co^2+^ cations with each facilitating the reduction of the other, likely due to a weakening of the interaction between isolated cations and the zeolite framework.

In bimetallic systems, Ni^2+^ and Co^2+^ cations compete for extra-framework cationic positions in the zeolite phases. As a result, these cations occupy positions where their interaction with the zeolite framework is diminished compared to monometallic system, facilitating their reduction at lower temperature.

### Catalytic test in citral hydrogenation

**Figure 5:**
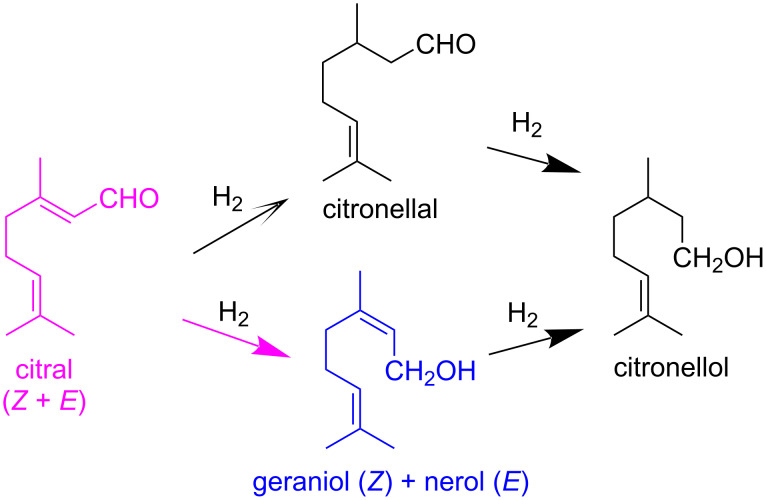
Main pathways of citral hydrogenation.

**Figure 6:**
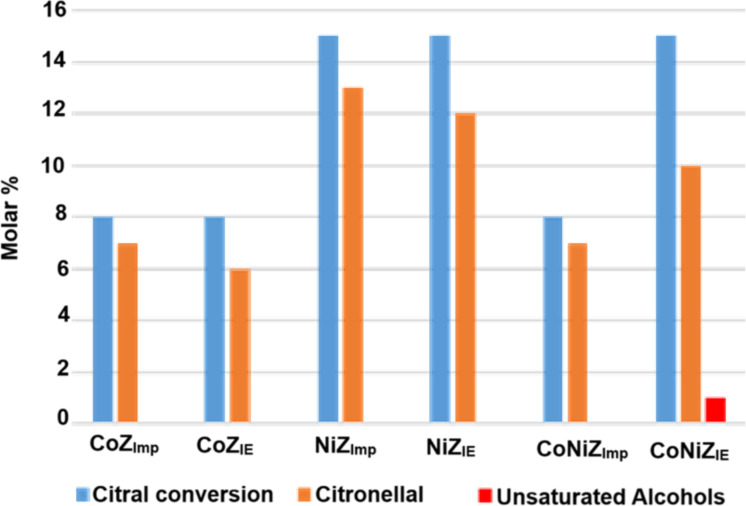
Citral hydrogenation results on the mono- and bimetallic catalyst materials obtained by IE and Imp.

The main pathway of citral hydrogenation is illustrated in Figure 5, and the corresponding results are presented in Figure 6. Catalysts were tested as obtained, that is, without prior thermal activation. Although the conversion of citral after 3 h of reaction is relatively low for all catalysts, it indicates that the active sites are accessible to citral molecules. Overall, citral hydrogenation and the formation of citronellal are higher for catalysts containing nickel compared to those containing cobalt (Figure 6). This suggests that the most active catalytic sites for the conversion of citral to citronellal are associated with nickel species. Notably, the bimetallic CoNiZ_IE_ catalyst is the only one to show activity for the hydrogenation of citral to the unsaturated alcohols geraniol and nerol (Figure 5), albeit in small quantities. In contrast, the bimetallic CoNiZ_Imp_ shows lower citral conversion, with no formation of unsaturated alcohols detected. This discrepancy between the bimetallic catalysts may be attributed to differences in ionic species, as well as variation in specific surface area (Table 3) and nickel content (Table 1 and Figure 3). The CoNiZ_IE_ catalyst has higher values for both parameters compared to CoNiZ_Imp_. Additional factors contributing to these differences will be discussed later.

The catalytic activity of the CoNiZ_IE_ catalyst in the selective hydrogenation of citral to unsaturated alcohols (geraniol and nerol) can be attributed to a synergistic interaction between cobalt and nickel species. These active species are likely associated with isolated cations or those formed during the hydrogenation process. For this reaction to proceed effectively, citral and hydrogen must interact directly with the active catalytic centers, such as isolated Ni^2+^ cations located in extra-framework cationic positions in the zeolite channels. Given that citral has a molecular diameter of around 0.3 nm, it can enter the channels of clinoptilolite (maximum opening of 0.31 × 0.75 nm) and mordenite (maximum opening of 0.70 × 0.65 nm), particularly the latter because of the larger channel diameter. The diffusion of reactants (citral and hydrogen) through the zeolite channels is expected to be favored by a higher specific surface area and unobstructed channel entrances. This is consistent with the superior surface area of CoNiZ_IE_ and the presence of impurities of mixed metal chloride salts on the surface of CoNiZ_Imp_, as evidenced by elemental analysis, XRD, and TPR profiles.

In order to improve these results, the CoNiZ_IE_ catalyst was subjected to in situ reduction at 500 °C for 2 h under H_2_ flow prior to the catalytic test. However, this high reduction temperature resulted to be deleterious to the catalytic performance. While the catalytic performance of CoNiZ_IE_ in the selective hydrogenation of citral to unsaturated alcohols remains low, the ability of this material to produce such alcohols indicates its potential. Future work will focus on optimizing the reduction temperature as lower reduction temperatures are expected to enhance performance. These findings will be addressed in subsequent studies.

## Conclusion

A detailed investigation was conducted on zeolitic materials featuring mono- and bimetallic systems of nickel and cobalt, derived from natural zeolite rich in clinoptilolite and mordenite. These materials were prepared using Ni^2+^ and Co^2+^ chloride solutions through traditional ion exchange (IE) and impregnation (Imp) methods. The Imp-prepared materials exhibited higher nickel and cobalt contents but also contained significant amounts of chlorides. Conversely, the IE-prepared materials showed negligible chloride content and larger specific surface areas. These differences were attributed to the substantial presence of chloride salts adsorbed on the surface of the Imp-prepared materials, as evidenced by XRD analysis. This also impacted the TPR profiles, with the Imp-prepared materials displaying hydrogen consumption patterns different from those of the IE-prepared materials. The TPR profiles further revealed that the thermal reduction of isolated Co^2+^ and Ni^2+^ ions (compensation cations in extra-framework ionic positions) was facilitated in the bimetallic systems, likely because of the synergetic interaction of multiple species and a reduced interaction between the cations and the zeolite framework. In line with these findings, the catalytic performance of the materials in the selective hydrogenation of citral showed marked differences. The most active catalytic sites for converting citral to citronellal were associated with nickel species. Among the catalysts, the bimetallic CoNi_IE_ material, prepared by IE, emerged as the most promising. It was the only catalyst to exhibit activity for the hydrogenation of citral to form unsaturated alcohols, suggesting a synergistic interaction between cobalt and nickel species. The active species are likely associated with isolated cations or those formed during the hydrogenation process.

This work highlights the potential of bimetallic CoNiZ_IE_ materials as efficient catalysts for selective hydrogenation reactions, paving the way for further optimization and exploration of similar catalytic systems.

## Experimental

### Material and methods

Natural zeolite from the San Andrés deposit in Cuba, with a particle size range of 40–160 μm, was used. This zeolitic material consists primarily of mordenite and clinoptilolite-type zeolites (around 80%), along with minor accompanying phases (quartz, montmorillonite, feldspar) [23]. It underwent a purification process following a method similar to one described previously [18]. Its elemental chemical composition in oxide form is 65.7% SiO_2_, 11.4% Al_2_O_3_, 3.4% CaO, 2.4% Na_2_O, 1.3% K_2_O, 1.1% MgO, and 2.6% Fe_2_O_3_. For simplicity, this purified zeolite is referred to as ZSA.

Monometallic (CoZ and NiZ) and bimetallic (CoNiZ) systems of nickel and cobalt were prepared from ZSA with NiCl_2_, CoCl_2_, and mixed NiCl_2_/CoCl_2_ solutions through both IE and Imp. The mixed NiCl_2_/CoCl_2_ solution was prepared by combining equal volumes of NiCl_2_ and CoCl_2_ solutions. The NiCl_2_ and CoCl_2_ used were reagent-grade, supplied by Sigma-Aldrich, St. Louis, MO, USA.

The IE processes were performed at 80 °C with reflux for 24 h, using a total of 2 milliequivalents of the corresponding cations (Ni^2+^, Co^2+^, and Ni^2+^/Co^2+^) in solution per gram of ZSA and 1 g/10 mL solid/solution ratio. The solid phases were washed with distilled water to remove chloride ions and oven-dried at 110 °C.

The Imp processes were conducted using a total of 6% of corresponding metals (Ni, Co, and Ni/Co) per gram of ZSA and solutions with total metal contents of 0.2 mol/L. Both the ZSA and the solutions were heated to 80 °C before being mixed. After 24 h, the solid phases were separated, lightly washed with distilled water, and oven-dried at 110 °C.

### Characterization

The elemental composition of ZSA and the modified materials from both treatments was determined using X-ray fluorescence analysis, performed with a ZETIUM PANalytical system. To characterize the specific surface area and pore structure of the materials, they were analyzed using Micro-Active software for TriStar II Plus instruments. Approximately 200 mg of each sample was degassed at 30 °C for 30 min and then at 250 °C for 4 h before surface area measurements via nitrogen adsorption at 77 K. The initial natural zeolite samples were also examined via powder X-ray diffraction (XRD) and scanning electron microscopy (SEM).

XRD patterns were recorded using a PW 1218 diffractometer (Philips, Almelo, Netherlands) equipped with a curved graphite monochromator and Cu Kα radiation (λ = 1.5406 Å). Data were collected at a scan speed of 2°/min with a step size of 0.05°. SEM images were acquired using a FEI Nova NanoSEM 450 electron microscope. For this purpose, samples were mounted on holders and coated with a thin layer of gold prior to observation.

Temperature-programmed reduction (TPR) analyses were performed on an AutoChem 2910 instrument (Micromeritics, USA) equipped with a thermal conductivity detector (TCD). The procedure for TPR involved heating the sample in a 1.0 vol % H_2_/Ar gas mixture at a flow rate of 30 mL/min, from room temperature to 600 °C, at a ramp rate of 5 °C/min. Hydrogen uptake was monitored using the TCD.

### Catalytic test in citral hydrogenation

In a manner analogous to [24], the hydrogenation of citral was conducted in a 250 mL autoclave equipped with a magnetic stirrer and a temperature control unit. The catalysts (400 mg) were immersed in 90 mL of isopropanol and transferred into the autoclave. The reactor was first purged with nitrogen and then with hydrogen before raising the temperature to 130 °C. A mixture of 3 mL citral and 10 mL isopropanol was then introduced into the reactor via a cylinder under 75 bar of hydrogen pressure. Time zero was considered at this point. During the catalytic test, the reaction was carried out under constant pressure using a pressure control system. After various reaction times, liquid samples were manually collected and analyzed by gas chromatography to determine conversion and selectivity values.

It is opportune to outline that preliminary experiments [25] conducted under various stirring conditions, catalyst loadings, and grain sizes confirmed the absence of both external and internal diffusion limitations.

## Supporting Information

File 1 Additional figures.

## Data Availability

Data generated and analyzed during this study is available from the corresponding author upon reasonable request.
