# Supplementary material for: Zeolite materials with Ni and Co: synthesis and catalytic potential in the selective hydrogenation of citral
Source: Beilstein J Nanotechnol. 2025 Apr 14;16:520–9. doi: 10.3762/bjnano.16.40 (PMC12018909; doi:10.3762/bjnano.16.40)
Supplement: File 1 — Additional figures. [file Beilstein_J_Nanotechnol-16-520-s001.pdf]

BEILSTEIN JOURNAL OF NANOTECHNOLOGY

Supporting Information

for

Zeolite materials with Ni and Co: synthesis and catalytic potential in the selective hydrogenation of citral

Inocente Rodríguez-Iznaga, Yailen Costa Marrero, Tania Farias Piñeira, Céline Fontaine, Lexane Paget, Beatriz Concepción Rosabal, Arbelio Penton Madrigal, Vitalii Petranovskii and Gwendoline Lafaye

Beilstein J. Nanotechnol. **2025,** *16,* 520–529. doi:10.3762/bjnano.16.40

Additional figures

License and Terms: This is a supporting information file under the terms of the Creative Commons Attribution License (https://creativecommons.org/licenses/by/4.0). Please note that the reuse, redistribution and reproduction in particular requires that the author(s) and source are credited and that individual graphics may be subject to special legal provisions.
The license is subject to the *Beilstein Journal of Nanotechnology* terms and conditions: (https://www.beilstein-journals.org/bjnano/terms)

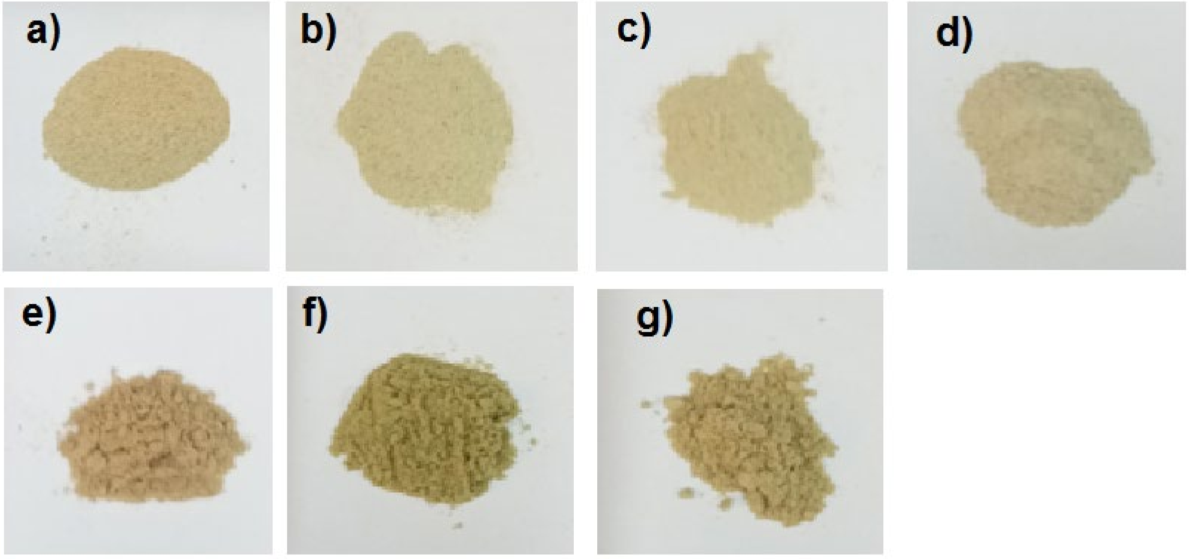

Figure S1: Photographs of samples of starting zeolite mineral (ZSA) and materials obtained through IE and Imp showing their color differences before and after treatments. (a) CoZ_{IE}, (b) NiZ_{IE}, (c) $CoNiZ_{IE}$, (d) ZSA, (e) CoZ_{Imp}, (f) NiZ_{Imp}, and (g) $CoNiZ_{Imp}$.

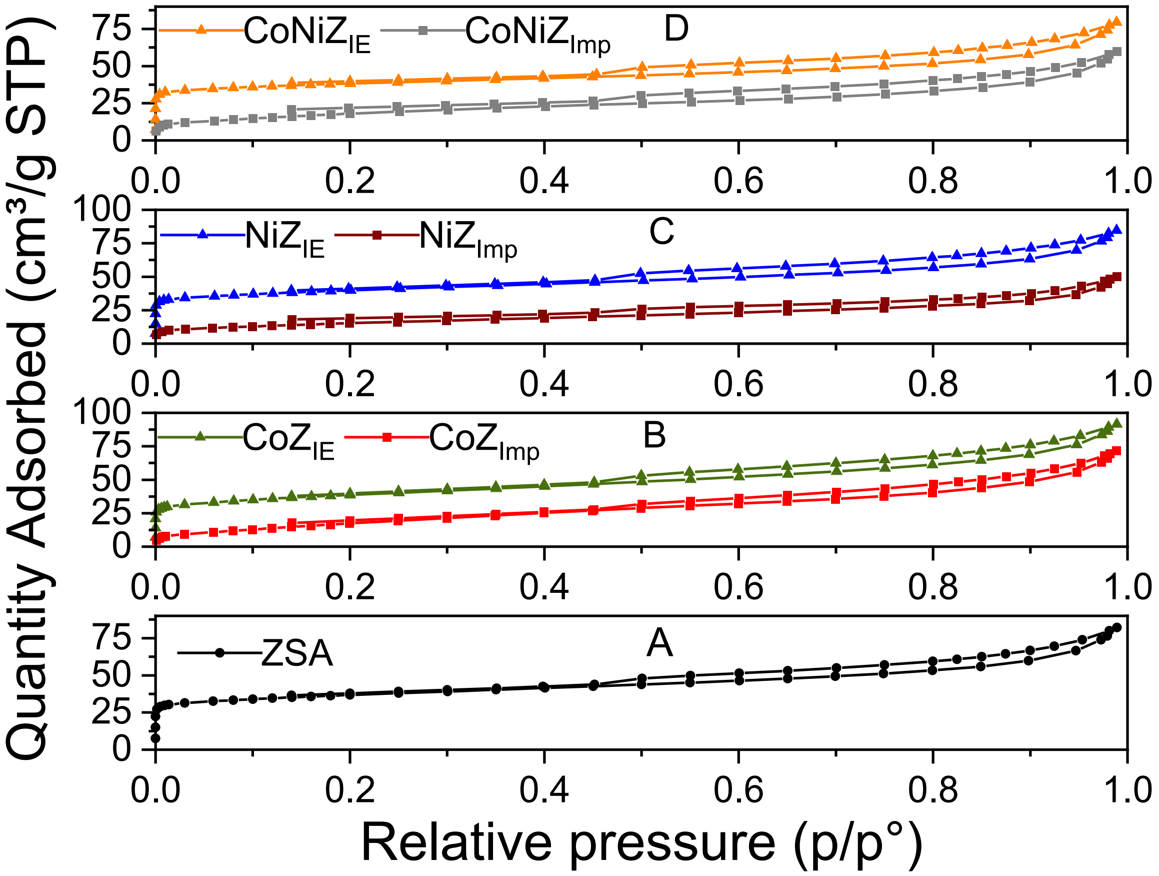

Figure S2: Adsorption isotherms of N_2 at 77 K for the starting zeolite mineral (A) and (B–D) materials obtained through IE and Imp.
